# Recurrent Pneumonia and a Normal Heart: Late Complication after Repair of Hemianomalous Pulmonary Venous Drainage—A Cautionary Tale

**DOI:** 10.1155/2010/930589

**Published:** 2010-03-09

**Authors:** Maryanne Caruana, Victor Grech, Jane Somerville

**Affiliations:** ^1^Department of Cardiology, Mater Dei Hospital, Birkirkara Bypass, Birkirkara MSD2090, Malta; ^2^Department of Paediatrics, Mater Dei Hospital, Birkirkara Bypass, Birkirkara MSD2090, Malta

## Abstract

Hemianomalous pulmonary venous drainage with intact atrial septum is a rare congenital anomaly and reports of its surgical repair and the long-term complications related to the correction are only infrequently encountered in the literature. We report the case of a patient with hemianomalous pulmonary venous drainage and intact atrial septum who underwent surgical repair using a pericardial baffle and creation of an “atrial septal defect” aged 15 years. Dyspnoea and recurrent chest infections started 7 months after surgery when he was seen by a respiratory physician without cardiac followup. He presented again aged 28 years with a recurrent pneumonia investigated over 6 weeks and heart pronounced normal from examination and echocardiography. Correct diagnosis was made in Grown Up Congenital Heart (GUCH) clinic stimulating review of data and catheterisation with pulmonary artery angiography which confirmed it. We feel that this case highlights the importance of specialist care and followup for GUCH patients.

## 1. Case Presentation

A gentleman born in 1979 had an asymptomatic murmur during childhood. Investigations confirmed hemianomalous pulmonary venous drainage to the right atrium without an atrial septal defect. The parents were Jehovah's witnesses and refused cardiac surgery with use of blood products. When aged 15 years, an adult cardiologist advised surgery and the patient was operated on by a general cardiac surgeon unused to congenital heart disease but willing not to use blood. The atrial septum was confirmed to be intact at operation and the oval fossa was enlarged to allow redirection of blood from the right pulmonary veins using a baffle of autologous pericardium to the left atrium. Recovery was uncomplicated. Seven months later he complained of increasing dyspnoea on effort and chest infections. He was reviewed once by a respiratory physician without further investigations or referral to cardiology. He trained and worked as a stonemason.

**Figure 1:**
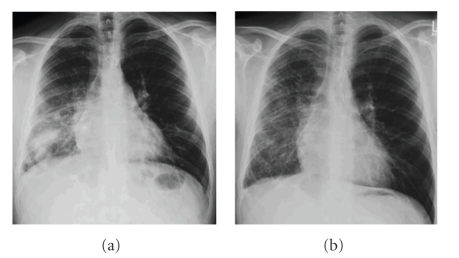
Chest radiographs (a) at presentation in GUCH clinic and (b) after several weeks of antibiotic treatment. Right pulmonary venous obstruction is suggested by the presence of dilated venules, Kerley lines and fluid in the fissures, all more obvious after the consolidation was treated.

He presented again aged 28 years, with a three-week history of right-sided pleuritic chest pain, high fever and a dry cough. On examination there was reduced air entry, coarse crepitations and a pleural rub at the right base. Chest radiography showed a dense patchy consolidation of the right lower lobe, probably also involving the middle lobe (Figure 1). He was treated with intravenous cefuroxime and oral clarythromycin but failed to respond as symptoms continued over several weeks with febrile exacerbations. An infectious disease specialist was involved and several alterations in the antibiotic regime were made. Tuberculosis was excluded on serial sputum cultures and infective endocarditis was considered possible but a transthoracic echocardiogram was reported as normal. Contrast computerised tomography scan of the thorax reported extensive consolidation within the right lung and probable chronic posterior pleural effusion. Bronchoscopy showed hyperaemic right main bronchial mucosa with inflammation especially around the right upper lobe bronchus. No lesions or pus were found to explain the situation. 

**Figure 2:**
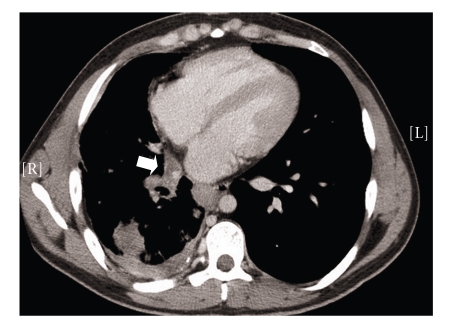
Contrast computerized tomography scan of the thorax showing right pulmonary venous obstruction (arrow), right lung consolidation and posterior pleural effusion.

Despite the “normality” of the heart but because of past cardiac surgery, he was referred to the monthly Grown Up Congenital Heart (GUCH) clinic. A diagnosis of obstructed right pulmonary venous drainage was made from the chest X-ray (Figure 1). This stimulated review of his previous investigations. Computerised tomography scan cuts at the level of the heart and pulmonary vessels showed completely obstructed right pulmonary veins (Figure 2). No pericardial baffle was identified upon review of cross-sectional transthoracic echocardiographic images. There was also no flow to be seen across the atrial septum at the level of the created “atrial septal defect” on colour Doppler. A repeat transthoracic echocardiogram with spectral Doppler interrogation of the pulmonary arteries showed normal antegrade flow from the main pulmonary artery into the left pulmonary artery but none into the right pulmonary artery.

A cardiac catheter study further confirmed no forward flow to the right lung at pulmonary artery wedge angiography, with normal flow to superior caval vein and left pulmonary artery. Pulmonary artery systolic pressure was 26 mmHg. After retrograde catheterisation of the left atrium, a baffle stump was seen but was found to be blind-ending and could not be crossed. Discussion of management with the GUCH Unit team at The Heart Hospital London concluded that it was unsuitable for intervention or operative reconstruction. The only treatment recommended was right pneumonectomy. The patient and family adamantly refused operation with use of blood and no surgeon agreed to perform it without blood as it is likely to be a very vascular operation. The patient has not had further infective recurrences but still complains of exertional dyspnoea. The family is searching for a surgeon who accepts their conditions.

## 2. Discussion

Partial anomalous pulmonary venous drainage with associated atrial septal defect is not an uncommon congenital malformation [1]. On the other hand, hemianomalous pulmonary venous drainage with intact atrial septum is a rare congenital anomaly [2, 3] often presenting as a simple atrial septal defect [1] and, as such, has only been reported in the literature on few occasions.

This case demonstrates points of clinical importance. The general cardiac surgeon who undertook the repair may not have been familiar with such a rare anomaly. Pulmonary vein obstruction is a known complication when pericardium is used in this way [4]. New chest symptoms and a history of cardiac surgery for congenital heart disease should have been correlated earlier. Presence of pulmonary venous obstruction is likely to have already been evident 7 months after his repair but unfortunately there was failure to have the patient adequately investigated. At this stage, it is likely that the lung was still salvageable and the obstruction might have been relievable. Yet at the time, the patient was not seen by any consultant with experience in congenital heart disease, particularly in adults or adolescents. The chest radiograph showed obvious Kerley lines as well as fluid in the fissures in keeping with pulmonary venous obstruction on the right, pointing to where and what to look for on computerised tomography of the thorax and on the echocardiogram.

This patient highlights the importance of having GUCH patients seen by specialists familiar with congenital heart problems [5]. This was an uncommon lesion repaired by a surgeon probably unfamiliar with the special techniques needed in such cases and not followed up with any informed evaluation. We caution those consultants in medicine, cardiology and general practice to seek specialist advice about patients with congenital heart disease.
